# Abnormality of Functional Connections in the Resting State Brains of Schizophrenics

**DOI:** 10.3389/fnhum.2022.799881

**Published:** 2022-03-10

**Authors:** Yan Zhu, Geng Zhu, Bin Li, Yueqi Yang, Xiaohan Zheng, Qi Xu, Xiaoou Li

**Affiliations:** ^1^College of Medical Instruments, Shanghai University of Medicine & Health Sciences, Shanghai, China; ^2^College of Medical Instrument and Food Engineering, University of Shanghai for Science and Technology, Shanghai, China; ^3^Shanghai Yangpu District Mental Health Center, Shanghai, China

**Keywords:** EEG, resting state, schizophrenia, functional brain network, machine learning

## Abstract

To explore the change of brain connectivity in schizophrenics (SCZ), the resting-state EEG source functional connections of SCZ and healthy control (HC) were investigated in this paper. Different band single-layer networks, multilayer networks, and improved multilayer networks were constructed and their topological attributes were extracted. The topological attributes of SCZ and HC were automatically distinguished using ensemble learning methods called Ensemble Learning based on Trees and Soft voting method, and the effectiveness of different network construction methods was compared based on the classification accuracy. The results showed that the classification accuracy was 89.38% for α band network, 82.5% for multilayer network, and 86.88% for improved multilayer network. Comparing patients with SCZ to those with Alzheimer’s disease (AD), the classification accuracy of improved multilayer network was the highest, which was 88.12%. The power spectrum in the α band of SCZ was significantly lower than HC, whereas there was no significant difference between SCZ and AD. This indicated that the improved multilayer network can effectively distinguish SCZ and other groups not only when their power spectrum was significantly different. The results also suggested that the improved multilayer topological attributes were regarded as biological markers in the clinical diagnosis of patients with schizophrenia and even other mental disorders.

## Introduction

The high morbidity and mortality of schizophrenia poses a serious impact and economic burden to society (Samsom and Wong, 2015). Nowadays, psychiatric patients are still diagnosed by experienced doctors through verbal communication and scale assessment (Kas et al., 2019). The diagnosis relies on symptomatic criteria and lacks objective biological indicators. Therefore, the search for landmark biological indicators has become an urgent breakthrough in psychiatric research.

The EEG originates from the cerebral cortex and reflects brain activity directly (Fornito et al., 2016). Resting-state EEG is recorded when a participant is awake and not engaged in any specific task, which can reflect the intrinsic ability of the brain (Duan et al., 2021). Most algorithms about EEG are interested in the time and frequency domain. In recent years, algorithms for analyzing the correlation of EEG signals from different channels have gradually become popular. The application of an EEG-based brain network has gained attention in the interconnected structure of the brain. Brain network techniques have been used to analyze the functional state of the brain in patients suffering from mental disorders, which are caused by structural damage or dysfunction of the brain. Zhao et al. (2021) captured abnormal brain changes in the SCZ by their tools for functional connectivity. Sun et al. (2017) extracted network features for evoked EEG of SCZ and HC with support vector machines (SVM) in which classification accuracy reached 80%, suggesting that the brain regions that play a major role were concentrated in the frontal and occipital lobes.

It is no doubt that EEG provides a reliable and useful method for understanding different psychiatric disorders (Newson and Thiagarajan, 2019). Certainly, EEG-based brain networks are different in patients with different psychiatric disorders. van Dellen et al. (2020) characterized functional connectivity and brain network characteristics in HC, SCZ, psychotic experiences, and treatment-naïve subclinical psychosis (SCP), and found that the functional networks of SCZ have many differences compared with other groups. Psychiatric disorders such as Alzheimer’s disease (AD) can be compared to each other by brain networks.

In essence, networks generally consist of nodes and edges between nodes (Barabási, 2013). Most EEG-based brain networks used electrodes to represent different brain regions as nodes to construct spatial networks. These constructed networks were mainly devoted to the analysis of the connections, distinctions, and the degree of lesions between different brain regions (Micheloyannis, 2012). In addition, the selection of electrode number is one of the most important elements for constructing EEG-based functional brain networks. EEG with more than 64 electrodes is recommended for the source connectivity method. However, the more the electrodes are used, the higher the clustering coefficient is generated, and it will be easier to generate weak connections and pseudo connections (Hassan and Wendling, 2018; Ismail and Karwowski, 2020). In contrast, some researchers argued that the use of EEG with fewer than 32 electrodes was better for monitoring brain activity. The data with a small number of electrodes (i.e.,≤16 channels) can be directly applied to clinical practice in a practical way (Wang et al., 2018; Li et al., 2019; Ismail and Karwowski, 2020). Racz et al. (2020) analyzed dynamic functional connectivity for the resting-state EEG with 19 electrodes of δ band in schizophrenics (SCZ), which used RF for classification to achieve a maximum cross validation accuracy up to 89.29%. Here, EEG signals with eight electrodes were used to study brain networks and to diagnose neurological diseases.

The edges of the network present the connectivity between nodes (Barabási, 2002). Various typical algorithms are available for defining edges in brain networks between two channels, such as mutual correlations for calculating the amplitude synchronization in the time domain (Wirsich et al., 2020), coherence for amplitude synchronization in the frequency domain (Zhang et al., 2020), phase-locking values (PLV), and phase-lag indices (PLI). Recently, PLV was widely used to study the connectivity of brain (Hassan and Wendling, 2018; Peng et al., 2019). Kim et al. (2020) constructed networks of EEG signals from different SCZ groups by PLV and the classification accuracy was 88.10%, which illustrated that SCZ and HC groups could be successfully classified by their network attributes.

The threshold selection is essential for building a powerless network. The appropriate threshold selection helps simplify the complexity of brain network computation by removing edges with low connection strength or other disturbances partly. On the contrary, the incorrect threshold selection causes instability and errors. According to the random graph model proposed by Erdõs and Rényi (1964), the minimum connection sparsity should be $2\frac{lnN}{N}$ to ensure full connectivity of the network, where *N* is the number of nodes. Combined with the definition of small-world networks, small-coefficient, σ, must be much larger than one when building a small-world network (Watts and Strogatz, 1998), which determines the upper limit of connection sparsity.

**FIGURE 1:**
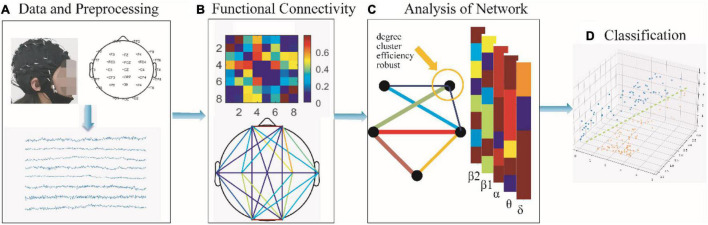
The framework proposed in this paper. **(A)** Collection of SCZ as well as HC resting-state EEG for 2 min followed by pre-processing. **(B)** Construction of single-layer brain networks with δ (0.5–4 Hz), θ (4–8 Hz), α (8–13 Hz), β1 (13–20 Hz), β2 (20–30 Hz), and multilayer brain networks. **(C)** Statistical analysis of networks, including the degree, clustering coefficient, local efficiency, and connectivity robustness. **(D)** Using classifiers for accurate discrimination of psychiatric disorders to find potential biological markers of SCZ depending on the classification results.

This paper proposes a framework for the analysis and classification of brain functional networks based on resting-state EEG together for applying on SCZ, as shown in Figure 1. The main contributions are summarized as follows:

(i) In this paper, the power spectrum of SCZ shows a significant increase in θ and α bands, and the results from the classification based on the single-layer network obtained by PLV–Filter–ELTS indicate that SCZ differs significantly in α and β1 bands from HC. It points to the possibility that information processing in the brain of SCZ may be abnormal from the perspective of energy changes, and the brain network attributes in the α band could be used as a potential biological marker.

(ii) The framework proposed enables to discover the results which cannot be obtained by non-network attributes (i.e., power spectrum analysis), illustrating that brain network attributes can be competitive candidates for biological markers in the clinical diagnosis of schizophrenia.

(iii) Applying data of SCZ and AD patients to the proposed framework, it turns out that the results of the multilayer network are more generalizable, which is not dependent on the power spectrum difference between the two groups.

## Materials and Methods

### EEG Recordings and Pre-processing

Twenty SCZ (8 women and 12 men, mean age 34.20 ± 4.74 years) and twenty AD patients (10 women and 10 men, mean age 65.25 ± 4.94 years) came from Shanghai Yangpu Mental Health Center. Twenty HC (9 women and 11 men, mean age 23.65 ± 3.31 years) had no personal history of neurological or psychiatric illness. All subjects involved in the research were screened by the Positive and Negative Syndrome Scale (PANSS), after providing informed consent. The Structured Clinical Interview for DSM-IV-TR (First et al., 2002) was administered by a psychiatrist (MJP) to assess psychiatric diagnoses of patients. Exclusion criteria for all subjects were identifiable neurologic disorders, substance use disorders within the last 6 months, or diagnosed sleep disorders. The study was approved by Shanghai Yangpu District Mental Health Center.

All experimental data were recorded in a quiet and closed room with no strong light, moderate temperature and humidity, good ventilation, and no electromagnetic interference. The subjects were kept awake and at resting state. EEG signals were recorded with a NeuroScan SynAmps2 Amplifier (Compumedics USA, Charlotte, NC, United States). The sampling rate was 1,000 Hz. Every electrode impedance was kept below 10 kΩ, and the electrodes were placed over the scalp according to international 10–20 system.

**FIGURE 2:**
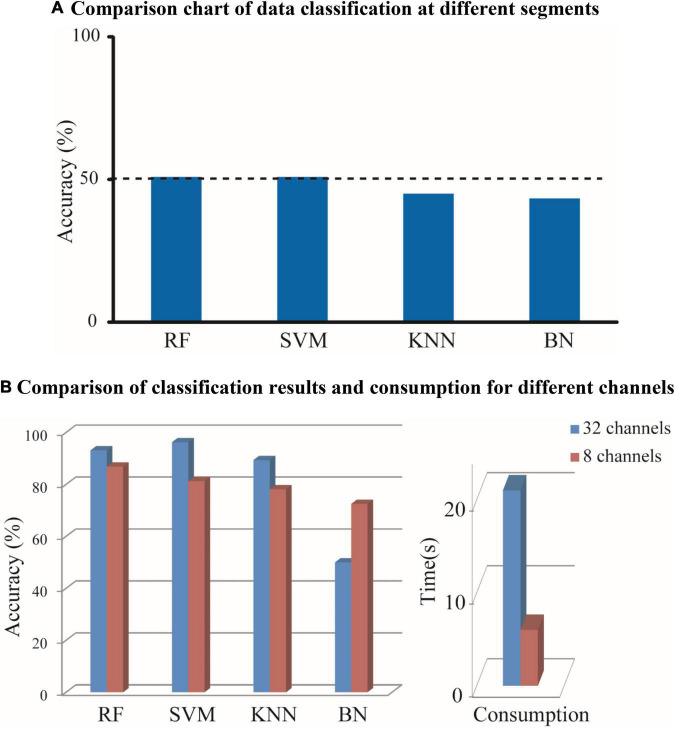
According to different consumption and a different number of channels, the EEG signals are recognized by a variety of classifiers. The classification accuracy was obtained and the consumption was calculated. **(A)** Comparison chart of data classification at different segments. **(B)** Comparison of classification results and consumption for different channels.

The EEG signals were average-referenced and bandpass-filtered with 0.1–30 Hz to obtain the desired frequency range and remove eye movements (Zhang et al., 2018). Furthermore, it was normalized to select 80-s data with high signal-to-noise ratio (Adamos et al., 2018). Each sample with 80 s was divided equally into four segments in the experiment. The first and second segments were classified with the third and fourth segments by SVM, K-Nearest Neighbor (KNN), Bayesian belief network (BN), and RF. The results obtained, as shown in Figure 2A, suggested that there was no significant difference, which indicates that the selected data are reasonable. Since the subsequent algorithm of functional connectivity (PLV) is only sensitive to phase and the number of our electrodes is low, it is not necessary with many pre-processing methods, and the volume conduction problem in EEG can be negligible.

Referring to the clinical recommendations of physicians, the EEG signal with 8-channel (FP1-2, C3-4, T3-4, O1-2) and the EEG signal with 32-channel after removing useless channels manually were constructed as brain network by PLV (threshold was chosen as 0.71, features were calculated as degree, clustering coefficient and local efficiency without extracting). As it is shown in Figure 2B, it is observed that the classification accuracy obtained with 8 channels is closer to those obtained with 32 channels, and the consumption with 8 channels can increase the running speed significantly compared with 32 channels. Thus, the EEG signal with 8-channel is selected for subsequent processing.

### Connectivity and Network Analysis

#### Functional Connectivity

The PLV was used to calculate the connection strength between nodes. Here, the instantaneous phase of a node (electrode) is calculated by wavelet transform. The PLV between electrode *x* and electrode *y* is $PLV=\left|\left\langle e^{i\theta_{xy}^{W}}\right\rangle \right|,\theta_{xy}^{W}=\theta_{x}^{W}\left(t\right)-\theta_{y}^{W}\left(t\right)$, where $\theta_{xy}^{W}$ is the phase difference between *x* and *y*, and *t* refers to the time.

**FIGURE 3:**
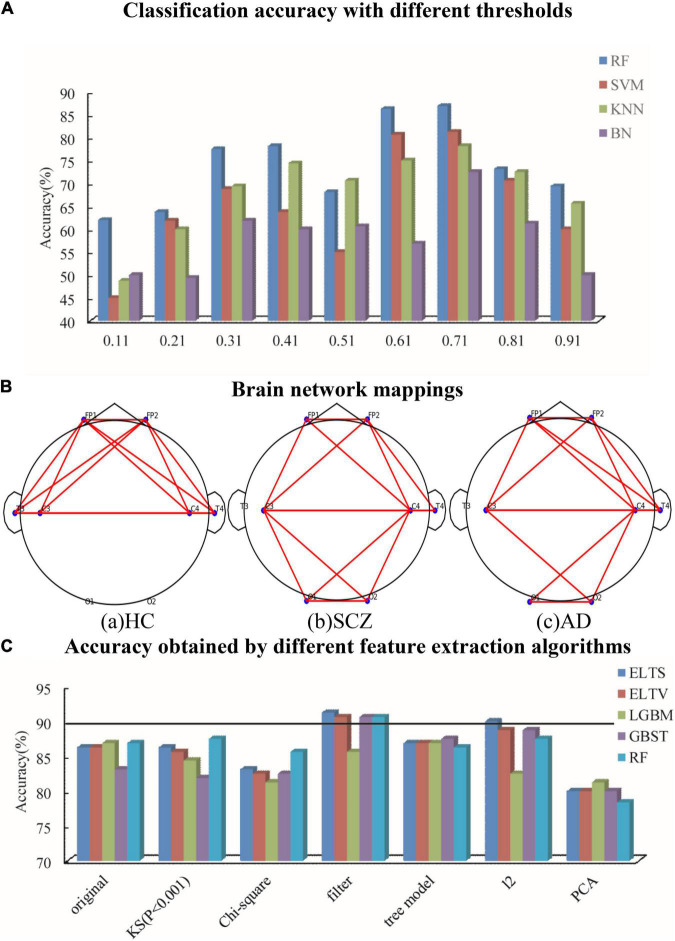
To find the best thresholds and features, it is classified for different threshold values and different feature selection methods. **(A)** Classification accuracy with different thresholds. **(B)** Brain network mapping. **(C)** Accuracy obtained by different feature extraction algorithms.

To find the best threshold in unweighted network, the threshold values of 0.11–0.91 are selected in steps of 0.1 to obtain the corresponding accuracy, which are listed in Figure 3A. The threshold is chosen as 0.71, which has the highest accuracy. The example mappings of brain networks for HC, SCZ, and AD patients are shown in Figure 3B.

#### Analysis of Single-Layer Network

The node degree *d_i_* of a node *i* is the number of edges connected to the node. The clustering coefficient measures the denseness of the network. In an unweighted network, the global clustering coefficient *C_i_* can be described as $\frac{d_{i}\left(d_{i}-1\right)}{2}$.

The local efficiency reflects the defense capability of the brain network, and to some extent the robustness of the brain network. It is calculated as follows $E_{i}=\frac{1}{N}$$\sum_{i\in G_{i}}\frac{1}{N_{G_{i}}\left(N_{G_{i}}-1\right)}\sum_{j\in G_{i},j\neq k}\frac{1}{l_{j,k}}$, where *G_i_* refers to the subgraph formed by the neighbors of node *i*, and *l*_*j,k*_ is the shortest path between nodes *j* and *k*.

The connectivity robustness refers to the strength of the remaining nodes after some nodes of the networks are damaged. Meanwhile, the importance of node *i* in the network can be understood by calculating the network attributes after removing node *i*. Dodds et al. (2003) proposed that the connectivity robustness *r_i_* is $\frac{d_{max}}{N-N_{i}}$, where *d*_*max*_ is the maximum degree value in the network after removing *N_i_* nodes. The connectivity robustness depends largely on degree distribution. Thus, to analyze the global attributes of brain networks, the connectivity robustness in this paper is calculated as $R_{i}=\frac{d_{avg}}{N-1}$, where *d*_*avg*_ is the average degree value in the network after removing node *i*.

#### Analysis of Multilayer Network

Multilayer networks fuse several single-layer networks, which allows the discovery of many hidden information that cannot be found by single-layer networks (Bianconi, 2018). In this paper, the multilayer networks based on EEG signals were constructed by different bands.

Buldyrev et al. (2010) proposed that the degree of node *i* in a multilayer network *D*_*mi*_ is $\sum_{a=1}^{M}D_{i}^{a}$, where $D_{i}^{a}$ is denoted as the degree of node *i* at layer *a* in the *M*-layer network. The clustering coefficients, local efficiency, and robustness of the multilayer network are defined as the following:


(1) $$\begin{array}{c} \left\{ \begin{array}{c} C_{mi}=\sum_{a=1}^{M}C_{i}^{a}\\ R_{mi}=\sum_{a=1}^{M}R_{i}^{a}\\ E_{mi}=\sum_{a=1}^{M}E_{i}^{a} \end{array}\right. \end{array}$$

During the calculation of the multilayer network, *D*_*mi*_, *C*_*mi*_, *R*_*mi*_, and *E*_*mi*_ are validated by combining the power spectrum in different frequency bands, which are different between patients that of HC. Based on the results shown in Figure 3, the power spectrum of different bands is used as the weight of the single-layer network as $D_{mi}=\sum_{a=1}^{M}\partial D_{i}^{a}$, where ∂ is the power spectral ratio of the network in that layer band. The power spectral density is estimated based on Welch periodogram method (Hamming window), with different values of ∂ for each sample. The clustering coefficients, local efficiency, and robustness of the improved multilayer network (IMN) are defined as follows:


(2) $$\begin{array}{c} \left\{ \begin{array}{c} C_{mi}=\sum_{a=1}^{M}\partial C_{i}^{a}\\ R_{mi}=\sum_{a=1}^{M}\partial R_{i}^{a}\\ E_{mi}=\sum_{a=1}^{M}\partial E_{i}^{a} \end{array}\right. \end{array}$$

### Extracts Important Features and Classification

Different classification algorithms were employed to distinguish network features from different groups (HC, SCZ, and AD), such as RF, SVM, K-nearest neighbor (KNN), and Bayesian belief network (BN). SVM uses the kernel function to map feature values to high-dimensional space, which is effective in solving small sample binary classification problems. KNN is a lazy learning algorithm for classification based on the distance between different feature values, which is suitable for the set of samples to be classified with more crossover or overlap. BN uses Bayesian formula to calculate the probability of the samples to be classified belong to each category, and finally selects the category based on the probability, which is simple in logic and easy to implement. As a newly emerged and highly flexible machine learning algorithm, RF consists of the final results of multiple decision trees, which allows for higher accuracy and generalization of the results.

Cross validation can repeatedly utilize samples to compose different training and testing sets to evaluate the goodness of model prediction, which is especially suitable for the case of small sample size. In this paper, the 10-fold cross-validation method was used to divide the training and test sets without repeated sampling, which can fully reduce the model overfitting phenomenon and improve the stability and generalization.

As shown in Figure 3A, comparing the results obtained by RF, SVM, KNN, and BN, it is suggested that the classification accuracy of RF has the highest accuracy and the most stable results. Therefore, tree models like RF are applied on the classification proposed.

The proposed classification is ELTS voting method, which is an ensemble learning method based on three tree models comprising of RF, light gradient boosting machine (LGBM), and gradient boosting survival tree (GBST). LGBM used the negative gradient of the loss function as the residual approximation of the current decision tree to fit the new decision tree, which is different from RF (Fan et al., 2019). GBST extends the survival tree models with a gradient-boosting algorithm, which is learned by minimizing the negative log-likelihood in an additive manner (Bai et al., 2021). The classification accuracy of RF, LGBM, GBST, Ensemble Learning based on Trees and Harder voting method (ELTH), and ELTS are listed in Figure 3C, which suggests that the classification accuracy of ELTS has the highest accuracy.

The data extracts important features through Kolmogorov–Smirnov Statistic (KS), Chi-square test, filter, tree model, *l*_2_ penalty term, and PCA. KS quantifies a distance between the empirical distribution function of two samples. In this paper, features are extracted from two samples when the empirical distribution function is lower than 0.001. What is different between KS and Chi-square test is that Chi-square test determines statistically significant differences by the expected frequencies and the observed frequencies from the two samples. Filter extracts important features based on the scores in various statistical tests and the various indicators of correlation. Tree model can extract the average of feature importance of all random trees and get the overall feature importance of the model, which can be used for feature selection and extraction. The operation of *l*_2_ penalty term in this paper is to combine *l*_2_ regularization and linear regression model, in other words, the *l*_2_ norm of coefficient *w* is added to the loss function as a penalty term when training the linear regression model, which forces the coefficients corresponding to those weak features to become 0 due to the non-0 regular term. Thus *l*_2_ penalty term becomes a good feature selection method. As the most commonly used data dimensionality reduction method, PCA reduces the data dimensionality while maintaining the features that contribute the most to the variance. The results are shown in Figure 3C, which shows that the highest accuracy is obtained by Filter–ELTS. Therefore, filter is selected to extract the more significant features before classification.

## Results

### Schizophrenics-Related Frequency Band

**FIGURE 4:**
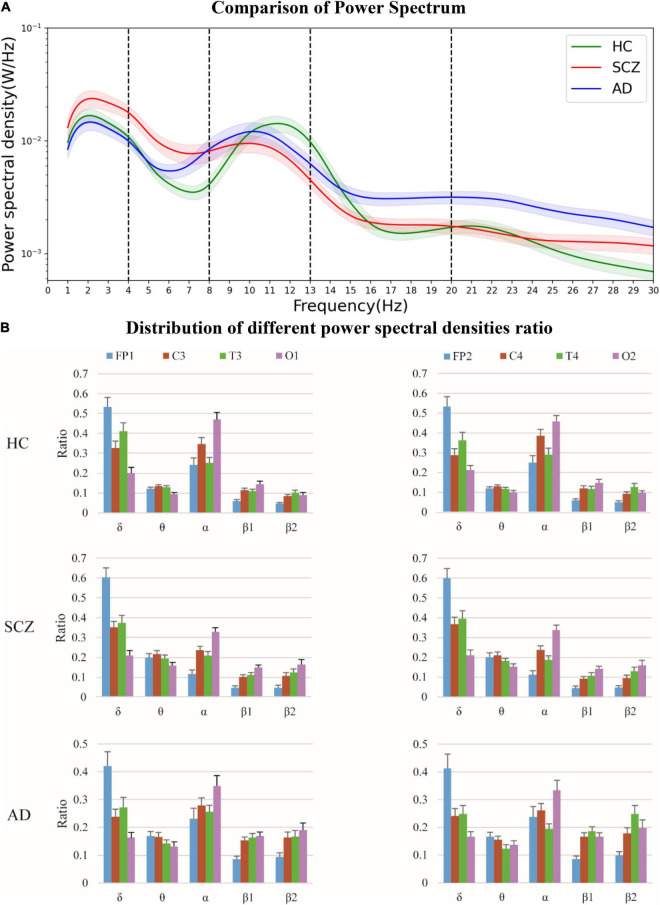
The power spectra were analyzed in HC, and in patients with SCZ, and AD, respectively, together with extracted power spectral density ratios of different brain regions. **(A)** Comparison of power spectrum. **(B)** Distribution of different power spectral densities ratio.

The comparison of the mean power spectra of all the channels for the three groups is shown in Figure 4A. Shaded area represents the standard error of mean. The difference between SCZ and HC is mainly concentrated between 0 and 16 Hz. The mean power spectra of SCZ is significantly higher in δ and θ bands and significantly lower in α band than that of HC. The power spectra of AD in θ band is less significant than that of SCZ, and the power spectra of AD in α and β bands is significantly higher than that of SCZ. Thus, it is illustrated that the power spectra in α band could be used as a potential biological marker between SCZ and HC.

The power spectral density ratios of the different channels are shown in Figure 4B. It suggested that the disparity in the prefrontal part (FP1-2) is more obvious in SCZ.

### Schizophrenics-Related Binary Network

**FIGURE 5:**
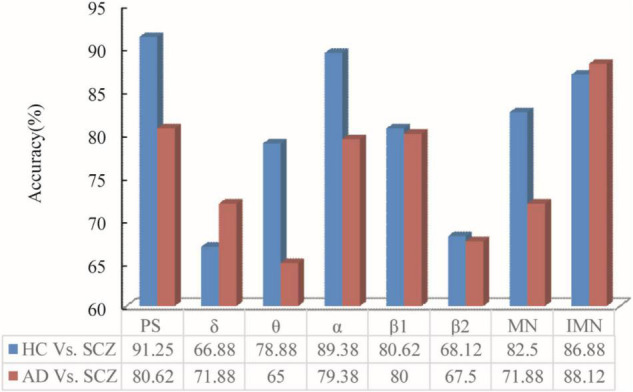
PS, δ, θ, α, β1, β2, MN, and IMN are used to classify by ELTS between the data of HC and SC. To verify the feasibility and generalizability, it was also applied to the data of AD patients and SCZ.

The power spectrum, single-layer networks in different bands, and multilayer networks are used to classify between SCZ and HC. The classification accuracy is listed in Figure 5, which indicate the classification accuracy of power spectrum (PS), single-layer network attributes (δ, θ, α, β1, and β2), original multilayer network attribute (MN), and IMN without δ and θ bands and taking into account the power spectral density ratio.

As can be seen from Figure 5, the classification accuracy obtained by PS–Filter–ELTS between SCZ and HC was as high as 91.25%. The classification accuracy according to the single-layer network attributes obtained from different bands showed that SCZ and HC had the highest classification accuracy in α band, indicating that the network attributes in α band were more different. Besides, the classification accuracy in both δ and θ bands was about 10% lower than those in α bands. Since the α and β bands of resting-state EEG in patients with mental illness have been the hotspot of research (Li et al., 2017; Zhang et al., 2021), the multilayer network in this paper only selected α, β1, and β2 bands to build the multilayer network while introducing power spectrum to adjust the parameters among each layer. The classification accuracy of IMN is up to 86.88%, which is 4.33% higher than MN.

### Generalization About the Proposed Framework

To verify the feasibility and generalizability, the proposed framework was also applied to the data of SCZ and AD patients. The classification accuracy between SCZ and AD is listed in Figure 5.

Between AD and SCZ, the accuracy of single-layer networks shows that accuracy in α band is 10% lower compared to the accuracy between SCZ and HC, which is in accordance with the power spectra that the difference in α band between AD and SCZ is not as obvious as between SCZ and HC. Moreover, it can be seen that the highest classification accuracy is 80.62% (PS) without using networks, and the highest classification accuracy is 88.12% (IMN) among all singe-layer networks and multilayer networks. The classification accuracy obtained from IMN was higher than that of PS, suggesting that brain network attributes are more generalizable between diseases or conditions in which the differences in α band are not obvious enough and brain network attributes can be explored further.

## Discussion

This paper shows that the power spectrum of SCZ increases significantly both in θ and α bands. According to the results of the single-layer network classification from the different bands obtained by PLV, Filter and RF, it is evident that SCZ differed from HC in the α and β1 bands. Both the non-network and network attributes suggest that abnormal brain function occurs in α band of SCZ. Similar results have been reported in several previous studies, such as Goldstein et al. (2015) proposed a defect in α-band activity in the EEG of SCZ, which was verified by Murphy and Öngür (2019). Newson and Thiagarajan (2019) reviewed various EEG studies of SCZ to point that SCZ had increased power in δ and θ bands and decreased power in α band, which was highly consistent with the findings of this paper.

The results from brain network attributes were different from those from non-network attributes (power spectra), suggesting the feasibility of exploring potential biomarkers between patients and HC using brain network attributes. A study combining brain network attributes and classification has been extensively explored. Jo et al. (2020) used the network attributes obtained from resting-state MRI data of SCZ and HC to classify by machine learning and verified that SCZ has impaired connectivity in the frontal–temporal–parietal regions.

The results of the proposed framework applied to the data of SCZ and AD patients estimated that the highest classification accuracy obtained by PS was 80.62%, and the highest classification accuracy of all singe-layer networks and multilayer networks was 88.12% (IMN). It means more generalizability of IMN–Filter–ELTS in diseases or between diseases where the difference in α band is not significant enough. Danjou et al. (2019) proposed that the variability in β and γ bands was more obvious in EEG of SCZ compared to EEG of AD patients during reviews of electrophysiological assessments between patients with SCZ and AD, which was in accordance with the findings of this paper.

The proposed framework is the classical “feature engineering + machine learning”; however, nowadays it is popular for “end-to-end” (Zeng et al., 2018). In contrast to those frameworks for “end-to-end,” “feature engineering + machine learning” can reflect the validity of features extracted through classification, which is exactly necessary to verify the importance of brain networks. In addition, since there are various features, it is no promise that the classification accuracy of a combination of multiple features can be higher than the single one. Hou et al. (2019) proposed a safe classifier that incorporates the idea of “ensemble learning,” which can solve this type of issue by combining multiple classical classifiers and different features. In this paper, the proposed classification also used an ensemble learning method based on three tree models comprising of RF, LGBM, and GBST by Soft voting method, which has a higher accuracy compared with RF, LGBM, and GBST. Furthermore, the study about improved classifiers will always be in progress.

The IMN–Filter–ELTS can be applied to the data between different diseases where the difference in α band is not significant enough, but it is also a challenge for the study of brain network attributes between different diseases with significant α band. It will be explored during pre-processing of EEG signals, functional connectivity of brain network, network attributes of brain network, classifier of different features, and other areas to solve this issue.

## Conclusion

In this paper, PLV is applied to construct the brain functional network of the resting-state EEG signals. The degree, local clustering coefficient, local efficiency, connection robustness, and the topology of the multilayer network constructed based on different bands are analyzed by statistical methods in the brain functional network. Moreover, the network attributes of both SCZ and HC are classified by ELTS.

Findings of brain network attributes implied that several significant differences exist between SCZ and HC, and machine learning can be used to appropriately classify patients with mental illness and HC as well as patients with different mental illness. Furthermore, the results of validating the proposed framework by data from AD suggest the generalizability of multilayer network attributes. It indicates that the multilayer network can be extended to diseases or disorders with insignificant differences in the α band, which has great research significance for the classification of a psychiatric patient.

## Data Availability Statement

The raw data supporting the conclusions of this article will be made available by the authors, without undue reservation.

## Ethics Statement

The studies involving human participants were reviewed and approved by the Shanghai Yangpu District Mental Health Center. The patients/participants provided their written informed consent to participate in this study.

## Author Contributions

YZ and GZ designed research and analyzed data. YZ, GZ, QX, XZ, and BL performed the research. YZ, GZ, XL, and YY wrote the manuscript. All authors contributed to the article and approved the submitted version.

## Conflict of Interest

The authors declare that the research was conducted in the absence of any commercial or financial relationships that could be construed as a potential conflict of interest.

## Publisher’s Note

All claims expressed in this article are solely those of the authors and do not necessarily represent those of their affiliated organizations, or those of the publisher, the editors and the reviewers. Any product that may be evaluated in this article, or claim that may be made by its manufacturer, is not guaranteed or endorsed by the publisher.
